# Decisional support needed when facing tough decisions: Survey of parents with children having differences of sex development

**DOI:** 10.3389/fruro.2023.1089077

**Published:** 2023-02-09

**Authors:** Melissa Gardner, William B. Brinkman, Meg Carley, Noi Liang, Sophie Lightfoot, Kendra Pinkelman, Phyllis W. Speiser, Tara Schafer-Kalkhoff, Kristina I. Suorsa-Johnson, Brian VanderBrink, Erica M. Weidler, Jessica Wisniewski, Dawn Stacey, David E. Sandberg

**Affiliations:** ^1^ Susan B. Meister Child Health Evaluation and Research Center, Department of Pediatrics, University of Michigan Medical School, Ann Arbor, MI, United States; ^2^ Division of General and Community Pediatrics, Cincinnati Children’s Hospital Medical Center, Cincinnati, OH, United States; ^3^ Department of Pediatrics, University of Cincinnati College of Medicine, Cincinnati, OH, United States; ^4^ Centre for Practice-Changing Research, Ottawa Hospital Research Institute, Ottawa, ON, Canada; ^5^ Patient/Parent/Caregiver Stakeholder Partners, Denver, CO, United States; ^6^ School of Nursing, University of Ottawa, Ottawa, ON, Canada; ^7^ Patient/Parent/Caregiver Stakeholder Partners, Ann Arbor, MI, United States; ^8^ Department of Pediatrics, Donald and Barbara Zucker School of Medicine at Hofstra/Northwell, Hempstead, NY, United States; ^9^ Institute of Molecular Medicine, Feinstein Institute for Medical Research, Manhasset, NY, United States; ^10^ Division of Endocrinology, Cincinnati Children’s Hospital Medical Center, Cincinnati, OH, United States; ^11^ Department of Pediatrics, University of Utah Spencer Fox Eccles School of Medicine, Salt Lake City, UT, United States; ^12^ Division of Urology, Cincinnati Children’s Hospital Medical Center, Cincinnati, OH, United States; ^13^ Division of Pediatric Surgery, Phoenix Children’s Hospital, Phoenix, AZ, United States; ^14^ Accord Alliance, Higley, AZ, United States; ^15^ Division of Pediatric Psychology, Department of Pediatrics, University of Michigan Medical School, Ann Arbor, MI, United States

**Keywords:** differences of sex development, disorders of sex development, intersex, decisional needs assessment, shared decision-making, decision-making preferences, decisional conflict

## Abstract

**Introduction:**

Parents of infants and young children newly diagnosed with differences of sex development (DSD) commonly face medical and psychosocial management decisions at a time when they are first learning about the condition and cannot consult their child for input. The aim of this study was to identify areas of greatest need for parental decisional support.

**Methods:**

34 parents of children receiving care for DSD at one of three US children’s hospitals participated in a survey to learn what clinical and psychosocial decisions needed to be made on behalf of their child. Parents were then asked to identify and focus on a “tough” decision and respond to questions assessing factors affecting decision-making, decision-making preferences, decisional conflict, and decision regret. Descriptive analyses were conducted.

**Results:**

Decisions about surgery and aspects of sharing information about their child’s condition with others were the two most frequently reported decisions overall, experienced by 97% and 88% of parents, as well as most frequently nominated as tough decisions. Many parents reported mild to moderate levels of decisional conflict (59%) and decision regret (74%). Almost all parents (94%) reported experiencing at least one factor as interfering with decision-making (e.g., “worried too much about choosing the ‘wrong’ option”). Parents universally reported a desire to be involved in decision-making – preferably making the final decision primarily on their own (79%), or together with their child’s healthcare providers (21%). The majority of parents judged healthcare providers (82%) and patient/family organizations (58%) as trustworthy sources of information.

**Discussion:**

Parents of children with DSD encounter medical, surgical, and psychosocial management decisions. Despite difficulties including emotional distress and informational concerns (including gaps and overload), parents express strong desires to play key roles in decision-making on behalf of their children. Healthcare providers can help identify family-specific needs through observation and inquiry in the clinical context. Together with families, providers should focus on specific clinical management decisions and support parental involvement in making decisions on behalf of young children with DSD.

## 1 Introduction

Defined as congenital conditions in which chromosomal, gonadal, and/or anatomic sex development is atypical, the umbrella term “disorders of sex development” (DSD)^a^(1) comprises a set of discrete diagnoses that present with a wide range of genital and reproductive anatomies (2, 3). Decisions, including those regarding gender of rearing, genetic testing, genital or gonadal surgery, and disclosure of the diagnosis, commonly arise (2–4).

Birth of a child with a DSD and uncertainties about the child’s physical health, gender, and psychosexual development are characterized as extraordinarily stressful (4, 5) and exert substantial strain on families (6, 7). In addition to healthcare decisions, challenges associated with having a child born with DSD can include changes in parental roles, responsibilities, goals, and social status (8). Parents describe both inadequate information and information overload, medical jargon and dismissive comments from healthcare providers, strong negative emotions (e.g., fear, guilt, uncertainty, shock, disbelief), and feeling overwhelmed (4, 8, 9), resulting in difficulty processing their child’s diagnosis (10). Parents worry that sharing information about their child’s condition will lead to rumors, gossip, and teasing; this, in turn, leads to isolation and withdrawal from usual support systems (4, 11, 12). Recognized by activists and providers, fear of stigmatization (6, 13–15) can precipitate parental decisions to surgically “fix” or “normalize” their child’s appearance before becoming fully informed about all options and properly weighing harms and benefits of surgery (16–19). Decisions involving genital surgery in young children, wherein parents reported being poorly informed or experiencing decisional conflict, were associated with decisional regret (20).

Despite such pressures, parents of children with DSD remain responsible for making decisions on behalf of their children and need to be able to participate in decision-making with clinicians. Following principles of patient- and family-centered care (a term intended to explicitly capture the importance of engaging the family and patient as essential healthcare team members) (21), the objective of shared decision-making (SDM) is to help make informed, preference-based clinical management choices among several relevant options (22). More specifically, SDM is a collaborative process where patients/parents and providers actively engage in healthcare decision-making by recognizing and acknowledging a decision is needed; discussing the best available evidence for each option, as well as their associated benefits and harms; and determining a preferred option by exploring patients’/parents’ informed preferences for option outcomes (23). Decision aids for healthcare decisions are tools that support SDM between patients/parents and providers by making the decision and treatment options explicit, providing evidence-based information about the associated benefits/harms, and helping patients to consider what matters most to them in relation to the possible outcomes (24).

Unmet parental decision-making needs may serve as barriers to applying SDM principles and use of decision aids. A recent study of healthcare providers identified several central “needs” of parents of children with a DSD: addressing parental distress and informational overload; information gaps including knowledge of the condition, options, and their features; and identification and incorporation of parental values for features of options (25). However, studies of decisional needs have not been conducted with parents of children with a DSD (26, 27). The aim of this study was to identify areas of greatest need for parental decisional support by assessing decisional experiences and preferences of parents of children with DSD.

## 2 Materials and methods

### 2.1 Study design

Parents of minor patients with DSD were recruited for participation in a web-based cross-sectional descriptive study between July and November 2021. The core Research Team convened a Steering Committee, comprised of those with complementary skills and experiences to those on the Research Team. Healthcare providers specializing in DSD (n=3), those directly affected by DSD (parent and/or adult patient; n=3, 2 of whom serve in patient advocacy leadership roles), and SDM or clinical researchers (n=6, inclusive of a member serving a dual provider/researcher role) comprised the full Research Team and Steering Committee. Including end users throughout the project ensured survey results would be relevant to them (28). The Research Team and Committee co-developed, conducted, and interpreted the findings of a survey focused on elucidating parental decisional needs. The study was guided by the Ottawa Decision Support Framework (ODSF) that conceptualizes the support needed by patients, families, and their healthcare providers for difficult decisions with multiple options whose features are valued differently (29). Recently updated after 20 years of use, the ODSF served to inform the survey development and analysis. Study protocol received ethical approval by the lead site’s Institutional Review Board (IRB), to which other sites’ IRBs had formally ceded oversight.

### 2.2 Participants and setting

Parents of children being seen for the assessment and/or ongoing clinical management of DSD at one of three medical centers with specialty DSD services were recruited. Patients served as index cases, not participants. DSD was defined following the 2006 Consensus Statement and 2016 Global Update definition: “congenital conditions within which the development of chromosomal, gonadal or anatomical sex is atypical” (2, 3); however, uncomplicated distal hypospadias, Klinefelter and Turner syndromes were excluded, as they do not present parents with the decisions commonly faced by parents of infants and young children with other DSD; e.g., genital and/or gonadal surgery. Case ascertainment lists were derived from electronic medical chart review using ICD-9 and -10 codes and DSD-related keywords (30). Patient age was limited to 18 and younger at the time of parent recruitment. Parent was broadly defined to include biological-, adoptive-, and step-parents, and other guardians who take active roles in managing their child’s condition. All eligible parents meeting those criteria with functional literacy in English were invited to participate.

Each of the three medical centers are children’s hospitals and members of the US-based Differences of Sex Development – Translational Research Network: a consortium of hospitals, medical centers, and a DSD-related non-profit advocacy and educational organization, working together to advance research, education, and clinical (31, 32). Sites regularly see patients aged newborn through young adulthood, with one having a greater focus on adolescent and young adult patients.

### 2.3 Recruitment and procedures

Participants were provided information about the study by research staff. For those whose clinic visits coincided with the recruitment window, recruitment was accomplished in person; otherwise, the initial contact was by phone. Individualized links to an online consent form and the survey were provided to those indicating interest in participation by email. Up to three reminders to complete the survey were sent after the initial email. Participants were provided a $20 honorarium for their time.

### 2.4 Materials

A survey, guided by the ODSF, focused on parental decisional needs was developed and included established (the Decisional Conflict (33), Decision-Making Preference (34), and Decision Regret (35) Scales) and novel measures (see **Supplementary Material**). The Decisional Conflict scale is a 16-item survey, scored 0 (no decisional conflict) to 100 (extremely high decisional conflict), with scores lower than 25 associated with implementing decisions and scores exceeding 37.5 associated with decision delay; test-retest and Cronbach alpha coefficients exceed 0.78; and the measure discriminates between known groups: those who make and delay decisions (effect size range 0.4 to 0.8) (33). The Decision-Making Preference scale assesses the degree of control desired in decision-making using a 5-point response scale, ranging from complete patient/parent control, through collaborative control, to complete healthcare provider control (34). The Decision Regret scale is a 5-item questionnaire; alpha coefficients range from 0.81 to 0.92; scale scores correlate with decision satisfaction (r’s=0.40 to -0.60) and decisional conflict (r’s = 0.31 to 0.52) (35); scores range from 0 to 100, with 0 indicating no decision regret, 1-25 mild regret, and >25 moderate to strong regret (36, 37).

The survey also included new items based on principles of SDM and decision aid development as informed by the ODSF, literature review, collective experiences of the Research Team and Steering Committee, and preliminary results of a recent survey of healthcare provider perspectives on parental decisional needs in DSD (25). After being asked what types of decisions parents have encountered related to their child’s DSD care, parents were asked to focus on a “tough” decision (a decision is tough when there is more than one option and none is clearly the best) and to describe it, including when it occurred. Subsequent survey items were completed in reference to that specific decision and the survey branched using wording to reflect whether the decision occurred in the past or was currently in the process of being made. The Decision Regret Scale and Decisional Conflict Effectiveness subscale were administered to those who indicated their tough decision had been made in the past. Following alpha testing to address functionality and usability, the survey was pilot tested with an adult living with DSD and two parents of a child with DSD focusing on user acceptance – with feedback provided both within free text blocks within the survey and verbally after survey completion.

### 2.5 Data analysis plan

Descriptive statistics (i.e., frequencies, percentages, means, standard deviations) are reported for quantitative items concerning parental decisional needs. Qualitative items (e.g., participant descriptions of tough decisions) were reviewed by three authors (MG, TS-K, EW) and coded for themes following a phenomenological approach (38) in which responses were read in their entirety, then salient themes were identified and mapped onto to the ODSF. Differences in coding were resolved through discussion that included KS-J and DES. In the one instance where two parents of the same index cases participated, data for both parents were retained given literature showing differences in experiences and needs for both in other pediatric health conditions (39, 40) and a preliminary review of data showing participants described different “tough” decisions experienced at different times in their child’s life.

## 3 Results

### 3.1 Participant characteristics

Of 63 parents invited to participate, 39 consented and 34 (54%) completed the survey, representing 33 index cases. Median age group was 30-39 years, most identified as women (n=29, 85%), White/Caucasian (30, 88%), married/partnered (27, 79%), and with a median household income of $80,000 - $100,000 (**Table 1**). Mean index case age at the time of recruitment was 11.2 ( ± 6.0) years; DSD diagnoses included congenital adrenal hyperplasia (n = 6, 18%), cloacal malformation/bladder exstrophy (6, 18%), complete or mixed gonadal dysgenesis (5, 15%), complete or partial androgen insensitivity syndrome (3, 9%), 17-beta-hydroxysteroid dehydrogenase type 3 deficiency (2, 6%), proximal hypospadias without genetic variant identified (2, 6%), and other individual conditions categorized as 46,XY, 46,XX (4, 12% each), or sex chromosome (1, 3%) DSD.

**Table 1:** Participant demographics.

	N	%
Gender		
• Women	29	85.3
• Men	5	14.7
Age (years)		
• 25-29	3	8.8
• 30-39	15	44.1
• 40-49	12	35.3
• 50-59	4	11.8
Race and Ethnicity		
• White/Caucasian	30	88.2
• African American/Black	1	2.9
• Asian	1	2.9
• Hispanic	1	2.9
• Prefer not to say	1	2.9
Marital Status		
• Married/Partnered	27	79.4
• Single	5	14.7
• Separated/Divorced	2	5.9
Household income		
• Less than $40,000	6	17.6
• $40,000 to less than $60,000	3	8.8
• $60,000 to less than $80,000	4	11.8
• $80,000 to less than $100,000	6	17.6
• $100,000 or more	12	35.3
• Prefer not to say	3	8.8

### 3.2 Types of decisions

Parents reported being faced with a number of decisions. The two most frequently reported were “Decisions about surgery” (n=33, 97%) and “Decisions about how much and when to share information about my child’s condition to extended family and close family friends” (30, 88%) (**Table 2**).

**Table 2:** Types of decisions made on behalf of children: Number (%) of parents making decisions about a topic.

Decision	All Decisions^a^		Tough Decisions^b^	
	n	%	n	%^c^
Surgery	33	97.1	18	52.9
How much and when to share information about my child’s condition to extended family and close family friends	30	88.2	9	26.5
How much and when to tell my child about their condition	25	73.5	4	11.8
Medication treatments	25	73.5	4	11.8
Seeing a mental health specialist for me or my child	25	73.5	0	0
Genetic testing	24	70.6	1	2.94
Other testing (for example, doing an examination with my child under anesthesia)	21	61.8	1	2.94
Attending a support group	15	44.1	0	0
Bringing my child up as a girl or boy	15	44.1	7	20.6

a Each “yes/no” item was asked of all participants; n and % reflect those answering “yes”.

b Participants listed and described a tough decision to focus on for the remainder of the survey.

c Nine (26%) parents reported multiple decisions resulting in summated response values greater than the number of participants and exceeding 100%.

### 3.3 Tough decisions

When asked to narrow the decisions to those categorized as “tough” and describe them, seven were identified: those concerning surgery (n=18, 53%); disclosing the child’s medical condition to friends, family and others (9, 26%); gender of rearing (7, 21%); educating the child about their condition and its management (4, 12%); medication treatment (4, 12%); and genetic or endocrine testing (2, 6%) (**Tables 2**, **3**). Nine (26%) parents reported more than one tough decision – some of which were overlapping. Common overlapping concerns were those regarding surgery and gender of rearing/gender identity (e.g., “Raising our daughter as male and not having surgery, [raising] her as female and having surgery”) and how much and when to tell extended family and friends as well as how much and when to educate the child about their own condition (e.g., “Not telling some family/friends. Also not telling our daughter until she is older, for fear she would tell others not understanding the consequences of her sharing.”). The majority (23, 68%) indicated the tough decision occurred in the past and 11 (32%) reported currently making the decision. Of those reporting past decisions, 3 (13%) occurred in within the last year, 5 (22%) 2-4 years ago, and 14 (61%) 5+ years ago; 1 (4%) reported not remembering when.

**Table 3:** Tough decisions: Themes and selected examples.

Themes	Selected Examples^a^
Surgery	• [First name] has severe hypospadias. We’re deciding to repair it now Or wait until he’s older. • In order for [First name] to have a sexual life with a partner of her choice, one would think she would need a vagina and which for that act to occur. However the surgery it’s self is very invasive and there are very few who have specialized in this kind of surgery so it is a difficult decision to make. • When to have the surgery
How much and when to share information with extended family and close family friends	• Everyone was aware that something was going on because of all of the medical appointments but what to tell people was the concern. Who would understand? Would we be able to answer the questions? Would they reject her? Would they tell others about her? • How, when, if to tell family/friends/classmates about our daughter’s condition. • This one above is a very difficult one for me personally beacause I have a fear of people treating my daughter differently, favoring her more than her brother because of all of it. Plus its her personally story and I don’t think everyone needs to know about it.
Bringing my child up as a girl or boy	• Our hardest decision was definitely on what gender to bring our child up as. We decided that female was the correct decision due to her genetically being female. • There was a lot of inner turmoil about choosing a gender for my child when I didn’t yet know how my child would identify. We also didn’t have much info on our daughter’s condition at the time. Not having access to current information and being told her condition was so rare that there weren’t many others in the US to consult with was very scary.
How much and when to tell my child about their condition	• When is a proper time to address our child’s condition? Do we express to him that he is perfectly made. That he is unable to bear his own children. • Age appropriate information and when is the right time to talk to my child so that he can understand
Medication treatments	• Throughout her childhood there were times when decisions had to be made about when was the best time to give her medication and how much - changing the dosage. • We were on a low dose of meds for the first few years of life, then at about age 4 her condition began to spiral and we had to decide about some different meds to get it back under control.
Genetic testing	• We understand that my husband and I are carriers of CAH and that is how my daughter ended up with the genetic disorder. However, I am wondering if genetic testing is necessary for her and my other children to see if they are also carriers or what information these tests will give to better support our family.
Other testing	• We were asked to draw about 10% of our son’s blood about 7 days after his birth and that was problematic. It was for testosterone measurements. We had many discussions and engaged the team and the actual lab for the testing. Due to that engagement, we were able to reduce the amount of blood that was actually needed for the test. It was a little traumatic considering our son was just born and the other issues that we were dealing with.
Bringing my child up as a girl or boy	• Our hardest decision was definitely on what gender to bring our child up as. We decided that female was the correct decision due to her genetically being female. • There was a lot of inner turmoil about choosing a gender for my child when I didn’t yet know how my child would identify. We also didn’t have much info on our daughter’s condition at the time. Not having access to current information and being told her condition was so rare that there weren’t many others in the US to consult with was very scary.
Include child^b^	• Have surgery within the next couple of years or allowing our child to decide when they are ready to have the surgery. • We have decided to wait until our child is ready to be part of the decision since they [ … ] are currently in the middle ground of knowing what would be happening but maybe not understanding it completely. • I felt very conflicted making a decision either way, as I ultimately didn’t believe the decision was mine to make.

a Selected examples comprise verbatim responses from participants in which typographical errors are preserved with one exception: to protect confidentiality, personally identifying information has been redacted and replaced by bracketed text.

b Contrasting with other themes, “include child” represents a decision-making preference identified in conjunction with tough decisions.

CAH, congenital adrenal hyperplasia.

### 3.4 Decisional conflict and decision regret

Regarding self-identified tough decisions, mean parental total decisional conflict was 28 (± 17) out of 100 (range = 0 to 65; **Table 4**), with no statistically significant differences between those responding in reference to a past decision (mean = 26 ± 16) versus a current one (mean = 30 ± 19), t(16) = 0.7, p<0.53. Mean subscale scores ranged between 21 and 44. Individual scores varied and 12 (35%) participants had significant decisional conflict exceeding scores of 37.5 out of 100 that is associated with decision delay. Fourteen (41%) had low decisional conflict with scores <25 out of 100.

**Table 4:** Parents’ decisional conflict.

		Past Decisions (n=23)		Current Decisions (n=11)	
		Mean	SD	Mean	SD
Total Decisional ConflictConflict Score^a^		26.2	15.6	30.5	19.5
Subscale Scores^b^					
Subscale name	Scale content				
• Uninformed	- *uninformed*	23.9	18.2	27.3	26.4
• Unclear values	- *unclear about personal values for benefits and risks/side effects*	22.5	17.8	26.5	20.7
• Unsupported	- *unsupported in decision-making*	33.3	27.3	24.2	19.9
• Uncertain	- *uncertainty about best choice*	33.0	19.7	43.9	30.5
• Effective Decision^c^	- *poor quality choice*	20.7	15.0	–	–

Measure scoring and interpretation are found in: O’Connor AM. User Manual – Decisional Conflict Scale^©^ 1993 [Updated 2010]. Available from: www.ohri.ca/decisionaid. Accessed December 22, 2022.

a Total score ranges from 0 = no decisional conflict to 100 = extremely high decisional conflict.

b Subscale scores ranges from 0 = no difficulty to 100 = extreme difficulty in each domain listed.

c The Effective Decision subscale was not administered to those currently making a decision.

With regard to individual scale items, the majority of parents disagreed with the statement that the decision was easy to make (22, 65%). Of those whose decision occurred in the past, additional top concerns included not having enough advice to make a decision (9, 39%) and not being able to choose without pressure from others (9, 39%). Of those currently making a tough decision, the majority (7, 64%) reported a lack of feeling sure about what to choose, followed by neither having enough advice (5, 46%) nor being clear about the best choice (5, 46%).

The mean decisional regret score among those whose decision occurred in the past was 18.5 out of 100 ( ± 15.6, range = 0 to 50), with most experiencing either mild (n=10, 43.5%) or moderate to strong (7, 30.4%) regret. There were no discernable patterns between decisional regret scores and specific tough decisions named by participants.

### 3.5 Factors contributing to difficulty in decision-making

The majority of parents (n=32, 94%) reported at least one factor that they experienced as contributing to difficulty in decision-making – including feeling “very emotional about [their] child’s condition” (n=26, 76%) and “too much [worry] about choosing the ‘wrong’ option” (20, 59%) (**Table 5**).

**Table 5:** Factors contributing to difficulty in decision-making.

	Past Decisions (n=23)		Current Decisions (n=11)	
	n	%	n	%
I felt very emotional about my child’s condition	19	82.6	7	63.6
I worried too much about choosing the “wrong” option	12	52.2	8	72.7
I felt too worried about others finding out about my child’s condition	10	45.5	3	27.3
I felt overloaded with information	10	45.5	3	27.3
It was hard to accept my infant’s diagnosis	9	39.1	3	27.3
I had difficulty discussing the decision with other family members	6	26.1	5	45.5
I did not have information on what others have decided	7	30.4	2	18.2
I did not have access to information on the condition and options	4	17.4	3	27.3
I did not have access to information on the benefits, harms, or other features of the options	4	17.4	3	27.3
We had difficulty discussing the decision as parents	2	9.1	3	27.3
I had difficulty discussing the decision with the health care providers	1	4.5	1	9.1
I was not invited to participate in decision-making	2	8.7	0	0
I did not have the skills to participate in making this type of decision	1	4.3	0	0

n and % reflect those answering “yes” to “yes/no/unsure” response options for each item.

Participants were free to skip individual items; percentages are calculated based on the number who answered individual items - which may differ from overall sample size.

For parents currently making a decision, items were phrased in the present tense (e.g., I feel…, I worry…, It is…).

These findings are also reflected in words parents used to describe the emotional strain associated with decisions (e.g., “inner turmoil” and “traumatic”), worries in the form of unanswered questions (e.g., “Who would understand? Would they reject her?” and “Do we express to him that he is perfectly made? That he is unable to bear his own children?”), and concerns over not having enough information (e.g., “I am wondering if genetic testing is necessary … or what information these tests will give” and “Not having access to current information … there weren’t many others in the US to consult with”) (**Table 3**).

Approximately half (46%) of past decision makers reported worrying about others learning about their child’s condition and feeling overloaded with information, compared with 27% of current decision makers. Conversely, approximately half (46%) of current decision makers reported difficulties discussing the decision with other family members, compared with 26% of past decision makers.

### 3.6 Peer support and resources involved in decision-making

With the exception of one parent, participants reported other people than themselves have been, or currently are, involved in making the tough decision. Most (n=30, 88%) involved their partner/spouse, followed by other family members (7, 30%), and friends (1, 3%). Seven (21%) parents also reported including their child (the patient). Aside from one parent who reported not accessing information and support resources when making decisions, citing “none really available to help make our decision,” most parents (n=28, 82%) reported using information provided by their healthcare provider during consultations to inform decisions, followed by contact with patient or family support groups (10, 29%), written resources (e.g., “materials provided to us by our clinic”; 6, 18%), and websites (5, 15%). Additional resources included self-directed web-based searches and information provided by friends, family, and older patients encountered at clinic visits or accessed through support groups.

**Figure 1:**
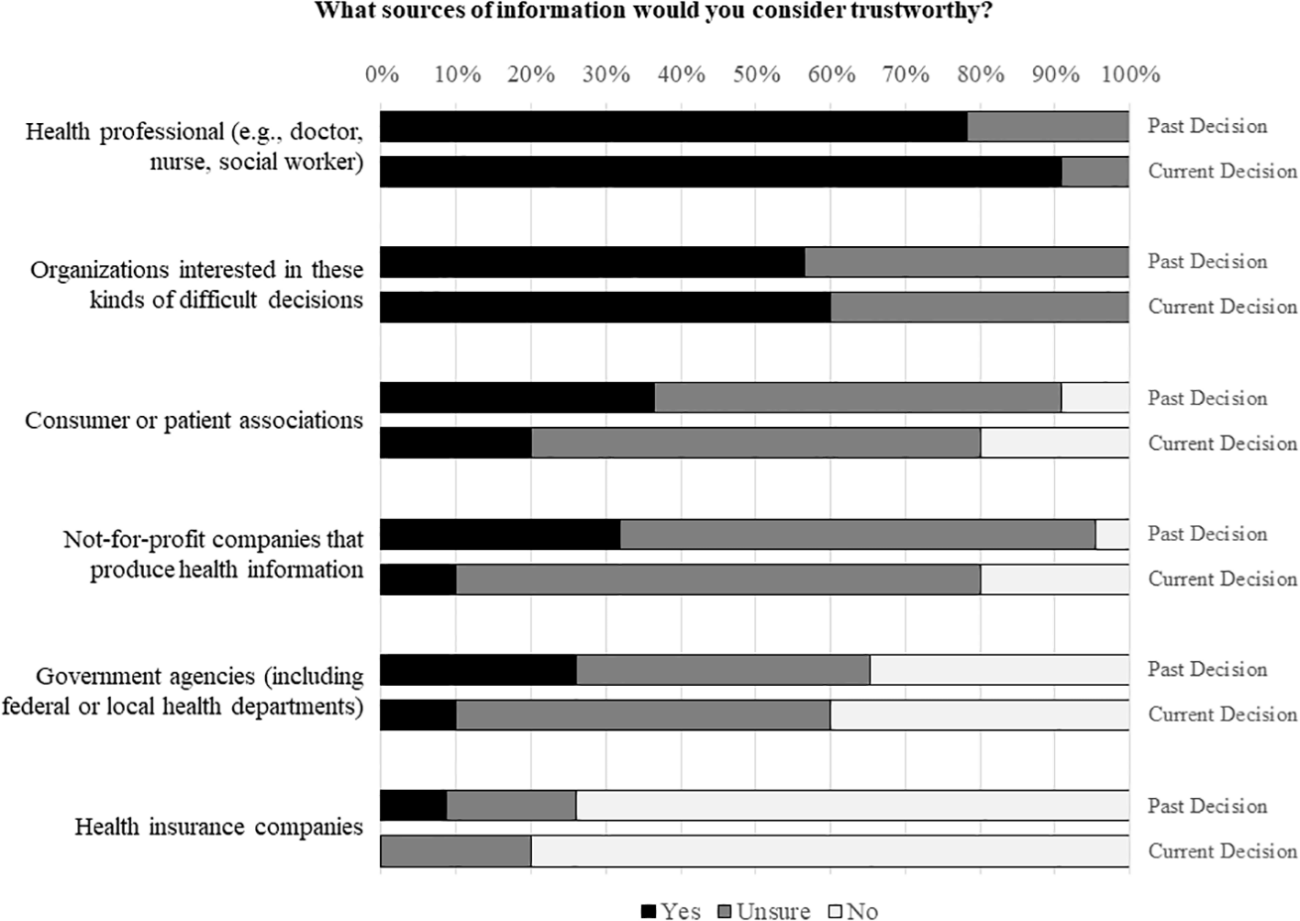
Parental trust in resources.

Parents reported varying degrees of doubt and uncertainty about the trustworthiness of some resources, including healthcare providers (n=6, 18% “unsure”; **Figure 1**). The majority of parents (25, 76%) did not consider health insurance companies as a trustworthy source; an additional six (18%) were unsure. Healthcare providers and organizations interested in these kinds of difficult decisions were rated as trustworthy by most parents (28, 82%; 19, 58%, respectively).

### 3.7 Decision-making preferences

Apart from one parent (5%) who reported that the tough decision was made entirely by the healthcare provider, most parents reported that either the final decision was one made mostly by themselves while strongly considering the provider’s opinion (n=12, 52%), entirely by themselves (6, 26%), or shared between themselves and the provider (4, 17%). A similar distribution of preferences was seen for those currently making a decision, with most (7, 64%) planning on making the decision themselves while strongly considering the provider’s opinion, (2, 18%) planning on making a shared decision, or (2, 18%) entirely by themselves.

For those who had already made their decisions, when asked how they would have preferred the decision be made if they had to do it all over again, parents reported preferences for arriving at the decision entirely by themselves (n=10, 45%), mostly by themselves with strong consideration of the healthcare provider’s perspectives (7, 32%), or together with the provider (5, 23%). No parents reported a preference for the provider to make the decision either entirely by themselves or mostly by themselves while considering parental perspectives.

In describing tough decisions they had made, four (12%) parents expressed a desire to include the child in decisions about surgery (n=3; e.g., “wait until our child is ready to be part of the decision”) and gender of rearing (n=1; e.g., “I ultimately didn’t believe the decision was mine to make”).

## 4 Discussion

### 4.1 Main findings

Our study identified several unmet decisional needs of parents with a child with DSD. Parents encountered several clinical management decisions on behalf of their children with DSD, with those concerning genital or gonadal surgery and discussions about the child’s condition and its management with others (including the child him/herself) most frequently mentioned. Decisions including those about genital or gonadal surgery, disclosure of the child’s condition to extended family and close friends, and gender of rearing were identified as particularly “tough” by parents. The majority of parents reported some level of decisional conflict and decision regret and almost all reported experiencing at least one factor as interfering with decision-making – most often emotions surrounding their child’s condition and worry about choosing the “wrong” option.

Compared with a recent report on healthcare providers’ perceptions (25), parents endorsed encountering these difficulties at rates lower than perceived by providers. Healthcare providers identified several common decisions encountered by parents, the first two described as particularly challenging: whether or not to choose genital or gonadal surgery, pursuing genetic testing beyond karyotype, accessing mental health services, and sharing details of the child’s DSD with relatives or close friends. While overlap between healthcare provider-report and our parent-report findings is apparent (e.g., genital or gonadal surgery; sharing details of the child’s condition), one-to-one correspondence was not observed, with only one (3%) parent reporting on genetic testing and none reporting on seeking mental health services as tough decisions. Our findings were consistent with a systematic review of decisional needs of parents making a wide range of healthcare decisions on behalf of their children that identified three key issues: need for information; talking to others (including concerns about pressure from others); and feeling a sense of control over the process that could be influenced by emotionally charged decisions, the consultation process, and structural or service (27).

A key feature of patient-centered care is meaningful patient and family participation in decisions about care (21, 41). Parents reported experiences of decision-making with healthcare providers that spanned the full range between exclusively parent-driven and exclusively provider-driven decisions. They universally reported a desire to be involved in decision-making – preferably making the final decision primarily on their own or, to a lesser extent, shared with their child’s healthcare providers. To be informed and effective participants in decision-making, parents require trustworthy resources of support and information – most often receiving information from providers and involving their partners in decision-making.

### 4.2 Strengths and limitations

Survey items primarily focused on the most difficult (“tough”) decisions identified as such by parents to concentrate our efforts at characterizing the features of these decisions, points at which parents experience the greatest decisional needs, and areas where clinicians and potentially others can intervene. Interpreting scale scores of measures indexing the decision-making process (e.g., levels of decisional conflict or regret) must be performed cautiously; such scores do not necessarily equally reflect the experience of the decision-maker across all clinical and psychosocial management issues. Approximately one third of parents reported currently making a “tough” decision; of those whose decision occurred in the past, over half reported decisions occurring five or more years ago, giving rise to the possibility of recall bias affecting scales requiring recollection of events (e.g., decisional conflict), but not those associated with current status (e.g., decisional regret).

DSD are individually rare conditions and our sample is relatively small. These limitations are balanced by the strength of a multisite recruitment strategy in which all eligible parents were invited to participate. Approximately half of those targeted participated: our sample was disproportionately women, White/Caucasian race, and non-Hispanic ethnicity. Notwithstanding the relative demographic homogeneity of our sample, the decision-making concerns and needs of our participants exhibited considerable variability. Indeed, such variability was seen between two parents of a single index case who identified different “tough” decisions experienced at different times. This speaks to a need for parents to be supported both individually and together in clinical practice.

### 4.3 Implications for care

Parents consistently voice a desire to take an active role in decision-making when decision points and treatment options are identified. Given variability in presenting features of the DSD requiring different decisions, parental decisional needs, and available social and information supports, clinicians are obliged to assess these for each patient (and family) in relation to each decision. High quality decision-making is often predicated on caregivers first resolving the tension experienced between the competing interests of promoting privacy versus establishing secrecy (42) regarding the child’s diagnosis and its implications which may bias decision-making in a manner that does not adequately take into account benefits and harms of all options. Healthcare providers can help identify family-specific needs through observation and inquiry in the clinical context

Some parents expressed a desire to include their child in decisions, but that their children were too young to be meaningfully involved. Healthcare providers can help parents by acknowledging the complexity of the situation and potential for challenges. Providers can help parents identify their own values and preferences and point our areas where differences between their own and those of their child may emerge over time.

### 4.4 Conclusions

Parents of children with DSD encounter medical, surgical, and psychosocial management decisions. Despite difficulties including emotional distress, worry, and informational concerns (including gaps and overload), parents express strong desires to play key roles in decision-making on behalf of their children. Healthcare providers should screen for and intervene on factors contributing to difficulty in the decision-making process. Educated and informed decision-making, supported by decision aids, can provide a robust mechanism for parent-provider collaboration within a patient- and family-centered care framework.

## Data availability statement

The datasets presented in this article are not readily available because the raw qualitative data cannot be sufficiently redacted to protect participant privacy and confidentiality. A dataset including the raw quantitative data supporting conclusions of this article will be made available. Requests to access the datasets should be directed to DES, dsandber@med.umich.edu.

## Ethics statement

The studies involving human participants were reviewed and approved by the University of Michigan Medical School - IRBMED. The participants provided their written informed consent to participate in this study.

## Author contributions

MG, WB, MC, NL, SL, KP, PS, KS-J, BV, JW, DS, and DES contributed to the conception or design of this work. MG, MC, SL, TS-K, KS-J, EW, DS, and DES contributed to the acquisition, analysis, or interpretation of data. MG and DES completed the original draft of this manuscript. All authors contributed to the critical revision of this manuscript, approved the version to be published, and agreed to be accountable for all aspects of the work. All authors contributed to the article and approved the submitted version.

## Footnotes

a The umbrella term “disorders of sex development” has been rejected by some patient advocates with concerns that the word “disorder” fosters stigma (1). For this reason, and following the practice of many others in this field, we have substituted “differences for “disorders” which preserves the acronym DSD.
