# Supplementary material for: Decisional support needed when facing tough decisions: Survey of parents with children having differences of sex development
Source: Front Urol. 2023 Feb 9;3:1089077. doi: 10.3389/fruro.2023.1089077 (PMC10621652; doi:10.3389/fruro.2023.1089077)
Supplement: Supplementary file 1 [file DataSheet_1.docx]

Supplementary Material

**Note: The following survey was programmed online. Item formatting followed that available in Qualtrics Survey Software. The survey branches in response to when the participants made a tough decision to follow either a past decision or a current decision path. Each participant’s own written response to Q2 (which decision would you like to focus on) was automatically piped into subsequent questions where *“[Q2]”* is typed into the item. Bracketed question numbers, measure names (e.g., Decisional Conflict Scale), and branching programming logic were not displayed to participants.**

**Title of Project:**Defining Successful Outcomes and Trade-Offs - Parental Decisional Needs

**Section 1: Decisions and Support**

In this section, we want to know your opinion about decisions that needed to be made for your child. In particular, we are interested in tougher health decisions.

| Definition of a tough decision:  A decision is tough when there is more than one option and none is clearly the best. For this type of decision, your choice will depend on your personal situation and you will have to weigh the pros and the cons of the possible options. Your choice should be informed by the best scientific information, but also by what is important to you and your family member (in other words, your preferred option). When faced with a tough decision, you may discuss it with a health provider (e.g. doctor, nurse, or social worker), family member or a friend. You may also read about it and take the time to consider what is best for your child. |
| --- |

**[Q1 : Types of Decisions]** What types of decisions have been made (or need to be made) for your child who has a condition affecting reproductive development? (*check all that apply*)

- Decisions about surgery
- Decisions about medications
- Decisions about genetic testing
- Decisions about other testing (for example, doing an examination with my child under anesthesia)
- Decisions about attending a support group
- Decisions about seeing a mental health specialist for me or my child
- Decisions about bringing my child up as a girl or boy
- Decisions about how much and when to share information about my child’s condition to extended family and close family friends
- Decisions about how much and when to tell my child about their condition
- Other decisions; *please describe*:

**We would like to ask you more about one of those decisions. Please choose one tough decision that you are willing to focus on for the next questions.**

**[Q2: Tough Decision]** Which decision would you like to focus on? *(please write-in):*

|  |
| --- |

**[Q2.1: Tough Decision]** Please describe this decision a little more. *(please describe):*

|  |
| --- |

**[Q3]** How long ago was the decision “*[Q2]*” made?

- I am currently making this decision
- Within the last 6 months
- 6 months to 1 year ago
- 2 to 4 years ago
- 5 or more years ago
- I don’t remember
- I prefer not to answer

[if Q3 = “I am currently making this decision,” a currently-making (cm) a decision version is substituted for selected questions.

Participants would only see and answer one version of the questions (either on the left side or the right side)]

| Past decision version | Currently-making (cm) a decision version – for selected questions |
| --- | --- |
| **[Q4: Tough Decision]** When you were thinking about the **options** for the tough decision “*[Q2]*”, what were the **options** you had to consider? (please describe):   \|  \| \| --- \| | **[Q4cm: Tough Decision]** As you think about the **options** for the tough decision “*[Q2]*”, what are the **options** you are considering? (please describe):   \|  \| \| --- \| |
| **[Q5]** Which option did you **choose**? (please describe):   \|  \| \| --- \| | [if Q3 = “I am currently making this decision,” Q5 is not administered] |
| **[Q6]** Was it your preferred option?   - Yes - No | [if Q3 = “I am currently making this decision,” Q6 is not administered] |
| **[Q7]** When you were thinking about the **options** for the tough decision “*[Q2]*”, were financial costs related to the options?   - Yes - No | **[Q7cm]** As you are thinking about the **options** for the tough decision “*[Q2]*”, are financial costs related to the options?   - Yes - No |
| **[Q7.1]** If yes, how did financial costs affect the decision? (please describe):   \|  \| \| --- \| | **[Q7.1cm]** If yes, how might financial costs affect the decision? (please describe):   \|  \| \| --- \| |

| **[Q8: Decisional Conflict Scale]** The following questions concern the tough decision “[*Q2*]”. Now think about the moment when you made your decision on behalf of your child, and please indicate to what extent you agree or disagree with the statements.   \|  \| Strongly Agree \| Agree \| Neither Agree nor Disagree \| Disagree \| Strongly Disagree \| \| --- \| --- \| --- \| --- \| --- \| --- \| \| 1. I knew which options were available \| □ \| □ \| □ \| □ \| □ \| \| 1. I knew the benefits of each option \| □ \| □ \| □ \| □ \| □ \| \| 1. I knew the risks and side effects of each option \| □ \| □ \| □ \| □ \| □ \| \| 1. I was clear about which benefits mattered most \| □ \| □ \| □ \| □ \| □ \| \| 1. I was clear about which risks and side effects mattered most \| □ \| □ \| □ \| □ \| □ \| \| 1. I was clear about which was more important (the benefits or the risks and side effects) \| □ \| □ \| □ \| □ \| □ \| \| 1. I had enough support from others to make a choice \| □ \| □ \| □ \| □ \| □ \| \| 1. I chose without pressure from others \| □ \| □ \| □ \| □ \| □ \| \| 1. I had enough advice to make a choice \| □ \| □ \| □ \| □ \| □ \| \| 1. I was clear about the best choice \| □ \| □ \| □ \| □ \| □ \| \| 1. I felt sure about what to choose \| □ \| □ \| □ \| □ \| □ \| \| 1. This decision was easy for me to make \| □ \| □ \| □ \| □ \| □ \| \| 1. I felt I had made an informed choice \| □ \| □ \| □ \| □ \| □ \| \| 1. My decision showed what is important \| □ \| □ \| □ \| □ \| □ \| \| 1. I expect to stick with this decision \| □ \| □ \| □ \| □ \| □ \| \| 1. I am satisfied with this decision \| □ \| □ \| □ \| □ \| □ \| | **[Q8cm: Decisional Conflict Scale]** The following questions concern the tough decision “[*Q2*]”. Now think about moments when you weigh your options on behalf of your child, and please indicate to what extent you agree or disagree with the statements.   \|  \| Strongly Agree \| Agree \| Neither Agree nor Disagree \| Disagree \| Strongly Disagree \| \| --- \| --- \| --- \| --- \| --- \| --- \| \| 1. I know which options are available \| □ \| □ \| □ \| □ \| □ \| \| 1. I know the benefits of each option \| □ \| □ \| □ \| □ \| □ \| \| 1. I know the risks and side effects of each option \| □ \| □ \| □ \| □ \| □ \| \| 1. I am clear about which benefits matter most \| □ \| □ \| □ \| □ \| □ \| \| 1. I am clear about which risks and side effects matter most \| □ \| □ \| □ \| □ \| □ \| \| 1. I am clear about which is more important (the benefits or the risks and side effects) \| □ \| □ \| □ \| □ \| □ \| \| 1. I have enough support from others to make a choice \| □ \| □ \| □ \| □ \| □ \| \| 1. I can choose without pressure from others \| □ \| □ \| □ \| □ \| □ \| \| 1. I have enough advice to make a choice \| □ \| □ \| □ \| □ \| □ \| \| 1. I am clear about the best choice \| □ \| □ \| □ \| □ \| □ \| \| 1. I feel sure about what to choose \| □ \| □ \| □ \| □ \| □ \| \| 1. This decision is easy for me to make \| □ \| □ \| □ \| □ \| □ \| |
| --- | --- | --- | --- | --- | --- | --- | --- | --- | --- | --- | --- | --- | --- | --- | --- | --- | --- | --- | --- | --- | --- | --- | --- | --- | --- | --- | --- | --- | --- | --- | --- | --- | --- | --- | --- | --- | --- | --- | --- | --- | --- | --- | --- | --- | --- | --- | --- | --- | --- | --- | --- | --- | --- | --- | --- | --- | --- | --- | --- | --- | --- | --- | --- | --- | --- | --- | --- | --- | --- | --- | --- | --- | --- | --- | --- | --- | --- | --- | --- | --- | --- | --- | --- | --- | --- | --- | --- | --- | --- | --- | --- | --- | --- | --- | --- | --- | --- | --- | --- | --- | --- | --- | --- | --- | --- | --- | --- | --- | --- | --- | --- | --- | --- | --- | --- | --- | --- | --- | --- | --- | --- | --- | --- | --- | --- | --- | --- | --- | --- | --- | --- | --- | --- | --- | --- | --- | --- | --- | --- | --- | --- | --- | --- | --- | --- | --- | --- | --- | --- | --- | --- | --- | --- | --- | --- | --- | --- | --- | --- | --- | --- | --- | --- | --- | --- | --- | --- | --- | --- | --- | --- | --- | --- | --- | --- | --- | --- | --- | --- | --- | --- |

| **[Q9: Factors Contributing to Difficulty in Decision-Making]** What factors made the decision “*[Q2]*” difficult?   \|  \| Yes \| No \| Unsure \| \| --- \| --- \| --- \| --- \| \| 1. It was hard to accept my infant’s diagnosis \| □ \| □ \| □ \| \| 1. I felt very emotional about my child’s condition \| □ \| □ \| □ \| \| 1. I worried too much about choosing the “wrong” option \| □ \| □ \| □ \| \| 1. I was not invited to participate in decision-making \| □ \| □ \| □ \| \| 1. I did not have access to information on the condition and options \| □ \| □ \| □ \| \| 1. I did not have access to information on the benefits, harms, or other features of the options \| □ \| □ \| □ \| \| 1. I did not have information on what others have decided \| □ \| □ \| □ \| \| 1. I felt overloaded with information \| □ \| □ \| □ \| \| 1. I felt too worried about others finding out about my child’s condition \| □ \| □ \| □ \| \| 1. I had difficulty discussing the decision with the health care providers \| □ \| □ \| □ \| \| 1. We had difficulty discussing the decision as parents \| □ \| □ \| □ \| \| 1. I had difficulty discussing the decision with other family members \| □ \| □ \| □ \| \| 1. I did not have the skills to participate in making this type of decision \| □ \| □ \| □ \| \| 1. Other   If *Yes* or *Unsure*, please describe (*write-in*): \| □ \| □ \| □ \| | **[Q9cm: Factors Contributing to Difficulty in Decision-Making]** What factors make the decision “*[Q2]*” difficult?   \|  \| Yes \| No \| Unsure \| \| --- \| --- \| --- \| --- \| \| 1. It is hard to accept my infant’s diagnosis \| □ \| □ \| □ \| \| 1. I feel very emotional about my child’s condition \| □ \| □ \| □ \| \| 1. I worry too much about choosing the “wrong” option \| □ \| □ \| □ \| \| 1. I am not invited to participate in decision-making \| □ \| □ \| □ \| \| 1. I do not have access to information on the condition and options \| □ \| □ \| □ \| \| 1. I do not have access to information on the benefits, harms, or other features of the options \| □ \| □ \| □ \| \| 1. I do not have information on what others have decided \| □ \| □ \| □ \| \| 1. I feel overloaded with information \| □ \| □ \| □ \| \| 1. I feel too worried about others finding out about my child’s condition \| □ \| □ \| □ \| \| 1. I have difficulty discussing the decision with the health care providers \| □ \| □ \| □ \| \| 1. We have difficulty discussing the decision as parents \| □ \| □ \| □ \| \| 1. I have difficulty discussing the decision with other family members \| □ \| □ \| □ \| \| 1. I do not have the skills to participate in making this type of decision \| □ \| □ \| □ \| \| 1. Other   If *Yes* or *Unsure*, please describe (*write-in*): \| □ \| □ \| □ \| |
| --- | --- | --- | --- | --- | --- | --- | --- | --- | --- | --- | --- | --- | --- | --- | --- | --- | --- | --- | --- | --- | --- | --- | --- | --- | --- | --- | --- | --- | --- | --- | --- | --- | --- | --- | --- | --- | --- | --- | --- | --- | --- | --- | --- | --- | --- | --- | --- | --- | --- | --- | --- | --- | --- | --- | --- | --- | --- | --- | --- | --- | --- | --- | --- | --- | --- | --- | --- | --- | --- | --- | --- | --- | --- | --- | --- | --- | --- | --- | --- | --- | --- | --- | --- | --- | --- | --- | --- | --- | --- | --- | --- | --- | --- | --- | --- | --- | --- | --- | --- | --- | --- | --- | --- | --- | --- | --- | --- | --- | --- | --- | --- | --- | --- | --- | --- | --- | --- | --- | --- | --- | --- |

**[Q10: Peer Support and Resources Involved in Decision-making]** Other than your child’s health care providers, who else has been involved in making the tough decision “*[Q2]*”? (*check all that apply*)

- Child (involved in the decision)
- My partner (husband/wife/other)
- Other member of immediate family, please specify (*write-in*):
- Friends
- Nobody, I made the decision alone.
- Other, please specify (*write-in*):

| Past decision version | Currently-making (cm) a decision version – for selected questions |
| --- | --- |
| **[Q11: Decision-making Preferences]** During the decision-making process for the difficult decision “*[Q2]*” you made, please indicate **how** you reached a decision: *(choose one)*   - I/we as the parents/caregivers made the decision - I/we as the parents/caregivers made the decision but strongly considered the health care provider’s opinion - I/we as the parents/caregivers and the health care provider made the decision together - The health care provider made the decision but strongly considered my/our opinion as the parents/caregivers - The clinician made the decision | **[Q11cm: Decision-making Preferences]** During the decision-making process for the difficult decision “*[Q2]*” you are making, please indicate **how** you plan to reach a decision: *(choose one)*   - I/we as the parents/caregivers will make the decision - I/we as the parents/caregivers will make the decision but strongly consider the health care provider’s opinion - I/we as the parents/caregivers and the health care provider will make the decision together - The health care provider will make the decision but strongly consider my/our opinion as the parents/caregivers - The clinician will make the decision |

| **[Q12: Decision-making Preferences]** If you had to do it all over again, how would you have preferred the decision be made? *(choose one)*   - I/we as the parents/caregivers make the decision - I/we as the parents/caregivers make the decision but strongly consider the health care provider’s opinion - I/we as the parents/caregivers and the health care provider make the decision together - The health care provider makes the decision but strongly considers my/our opinion as the parents/caregivers - The clinician makes the decision | [if Q3 = “I am currently making this decision,” Q12 is not administered] |
| --- | --- |
| **[Q13: Peer Support and Resources Involved in Decision-making]** What information and support resources did you use when make these decisions? (*check all that apply*)   - Information the health provider gave me/us during the consultation - Written resources; if yes, please give examples: - Patient / family support groups; if yes, please describe: - Websites; if yes, please describe: - Other; if yes, please describe: | **[Q13cm: Peer Support and Resources Involved in Decision-making]** What information and support resources are you using to make these decisions? (*check all that apply*)   - Information the health provider gave me/us during the consultation - Written resources; if yes, please give examples: - Patient / family support groups; if yes, please describe: - Websites; if yes, please describe: - Other; if yes, please describe: |

| **[Q14: Decision Regret]** Please think about the tough decision “[*Q2*]” that was made. Indicate whether you agree or disagree with the following statements by choosing which one best represents your opinion regarding this decision.   \|  \| Strongly Agree \| Agree \| Neither Agree Nor Disagree \| Disagree \| Strongly Disagree \| \| --- \| --- \| --- \| --- \| --- \| --- \| \| 1. It was the right decision \| □ \| □ \| □ \| □ \| □ \| \| 1. I regret the choice that was made \| □ \| □ \| □ \| □ \| □ \| \| 1. I would go for the same choice if I had to do it over again \| □ \| □ \| □ \| □ \| □ \| \| 1. The choice did a lot of harm to my child \| □ \| □ \| □ \| □ \| □ \| \| 1. The decision was a wise one \| □ \| □ \| □ \| □ \| □ \| | [if Q3 = “I am currently making this decision,” Q14 is not administered] |
| --- | --- | --- | --- | --- | --- | --- | --- | --- | --- | --- | --- | --- | --- | --- | --- | --- | --- | --- | --- | --- | --- | --- | --- | --- | --- | --- | --- | --- | --- | --- | --- | --- | --- | --- | --- | --- | --- |

**[Q15: Peer Support and Resources Involved in Decision-making]** What sources of information would you consider trustworthy? (*check all that apply*)

- Organizations interested in these kinds of difficult decisions (e.g. …)
- Health professional (e.g. doctor, nurse, social worker)
- Government Agencies (including federal or local health departments)
- Health insurance companies
- Consumer or patient associations (e.g., …)
- Not-for-profit companies that produce health information
- Other, please specify:

**[Q16]** If there is anything that you would like to share with us that was not covered in the items, please let us know:

| (write in): |
| --- |

**Section 2. About you**

Please tell us a bit about yourself:

In what age category are you?

- Under 25 years old
- 25-29
- 30-39
- 40-49
- 50-59
- 60 +
- Prefer not to specify

Your gender

- Man
- Woman
- Other; please describe:
- Prefer not to say

What is the highest grade/level of education you have completed?

- Less than 7th grade
- Completed junior high, including the 9th grade
- Partial high school, 10th or 11th grade
- High school graduate
- Partial college or at least one year of specialized training
- Standard college or university graduation
- Graduate/professional training
- Prefer not to say

In which state do you live? [drop down list]

People come from many different cultural and racial backgrounds.

What is your race? (*check all that apply*)

- African American/Black
- Asian
- First Nations/American Indian or Alaskan Native
- Hawaii Native/Pacific Islander
- Hispanic
- White/Caucasian
- Other: please specify: ________________
- Prefer not to say

Are you a member of any of the following groups? (*check all that apply*)

- Gender-diverse (e.g., agender, non-binary, transgender)
- Intersex
- LGBTQ+ (lesbian, gay, bisexual, transgender, queer, questioning)
- Neurodivergent (e.g., ADHD, autistic, dyslexic)
- Disabled, or caregiver of a person with a disability
- Member of a marginalized group not listed; please specify: ____________
- None of the above
- Prefer not to say

What is your marital status?

- Single
- Married / Partnered
- Separated/Divorced
- Widowed
- Other (please describe):
- Prefer not to say

Which of the following categories best describes your household income, before taxes and other deductions, for the last 12 months?

- Less than $40,000
- $40,000 to less than $60,000
- $60,000 to less than $80,000
- $80,000 to less than $100,000
- $100,000 or more
- Prefer not to say

Thank you. That is the last question we have for you.
